# Comparison and Characterization of Phenotypic and Genomic Mutations Induced by a Carbon-Ion Beam and Gamma-ray Irradiation in Soybean (*Glycine max* (L.) Merr.)

**DOI:** 10.3390/ijms24108825

**Published:** 2023-05-16

**Authors:** Zhuo Feng, Yan Du, Jingmin Chen, Xia Chen, Weibin Ren, Lulu Wang, Libin Zhou

**Affiliations:** 1 Biophysics Group, Biomedical Center, Institute of Modern Physics, Chinese Academy of Sciences, Lanzhou 730000, China; 2 University of Chinese Academy of Sciences, Beijing 100049, China; 3 College of Life Science and Technology, Gansu Agricultural University, Lanzhou 730070, China

**Keywords:** soybean, carbon-ion beam, gamma rays, mutation screening, whole-genome sequencing, structural variation

## Abstract

Soybean (*Glycine max* (L.) Merr.) is a nutritious crop that can provide both oil and protein. A variety of mutagenesis methods have been proposed to obtain better soybean germplasm resources. Among the different types of physical mutagens, carbon-ion beams are considered to be highly efficient with high linear energy transfer (LET), and gamma rays have also been widely used for mutation breeding. However, systematic knowledge of the mutagenic effects of these two mutagens during development and on phenotypic and genomic mutations has not yet been elucidated in soybean. To this end, dry seeds of Williams 82 soybean were irradiated with a carbon-ion beam and gamma rays. The biological effects of the M_1_ generation included changes in survival rate, yield and fertility. Compared with gamma rays, the relative biological effectiveness (RBE) of the carbon-ion beams was between 2.5 and 3.0. Furthermore, the optimal dose for soybean was determined to be 101 Gy to 115 Gy when using the carbon-ion beam, and it was 263 Gy to 343 Gy when using gamma rays. A total of 325 screened mutant families were detected from out of 2000 M_2_ families using the carbon-ion beam, and 336 screened mutant families were found using gamma rays. Regarding the screened phenotypic M_2_ mutations, the proportion of low-frequency phenotypic mutations was 23.4% when using a carbon ion beam, and the proportion was 9.8% when using gamma rays. Low-frequency phenotypic mutations were easily obtained with the carbon-ion beam. After screening the mutations from the M_2_ generation, their stability was verified, and the genome mutation spectrum of M_3_ was systemically profiled. A variety of mutations, including single-base substitutions (SBSs), insertion–deletion mutations (INDELs), multinucleotide variants (MNVs) and structural variants (SVs) were detected with both carbon-ion beam irradiation and gamma-ray irradiation. Overall, 1988 homozygous mutations and 9695 homozygous + heterozygous genotype mutations were detected when using the carbon-ion beam. Additionally, 5279 homozygous mutations and 14,243 homozygous + heterozygous genotype mutations were detected when using gamma rays. The carbon-ion beam, which resulted in low levels of background mutations, has the potential to alleviate the problems caused by linkage drag in soybean mutation breeding. Regarding the genomic mutations, when using the carbon-ion beam, the proportion of homozygous-genotype SVs was 0.45%, and that of homozygous + heterozygous-genotype SVs was 6.27%; meanwhile, the proportions were 0.04% and 4.04% when using gamma rays. A higher proportion of SVs were detected when using the carbon ion beam. The gene effects of missense mutations were greater under carbon-ion beam irradiation, and the gene effects of nonsense mutations were greater under gamma-ray irradiation, which meant that the changes in the amino acid sequences were different between the carbon-ion beam and gamma rays. Taken together, our results demonstrate that both carbon-ion beam and gamma rays are effective techniques for rapid mutation breeding in soybean. If one would like to obtain mutations with a low-frequency phenotype, low levels of background genomic mutations and mutations with a higher proportion of SVs, carbon-ion beams are the best choice.

## 1. Introduction

Soybean (*Glycine max* (L.) Merr.) is one of the most economically and nutritionally valuable crops in the world [1]. It is also the main source of protein in animal feed, as it is composed of essential amino acids and energy. About 70% of the protein consumed comes from soybean; thus, it plays an important role in the feed market [2]. High-protein alleles are mostly sourced from soybean plants that have been introduced [3], and they may be used as a source of genetic diversity in breeding programs for important traits [4]. Genetic variation, which can provide a variety of alleles, is an important source of plant improvement and adaptation. In plant breeding, the development of new traits also requires abundant alleles. The application of mutation techniques, including those using physical mutagens, chemical agents and biological techniques, has created many genetic variations. However, the differences among different physical mutagens in breeding are still unclear.

The genome sequence of the soybean MG III cultivar, which is named “Williams 82”, was published in 2010 [5]. The first soybean genome size was estimated at about 1.1 Gb. The soybean genome data file was approximately 955 Mb, and recently it was revised to version 4.0 with a size of 978.4 Mb [5]. The genome sequence was used to detect copy number variations (CNVs) using an array of comparative genomic hybridization (CGH) platforms [6]. Whole-genome sequencing (WGS), which is a powerful tool for researchers, is considered to be an effective method for the detection of single-nucleotide variations [7]. Recently, WGS has been widely used to analyze mutant spectra at the whole-genome scale and to map causal mutations.

Structural variants (SVs), such as deletions, insertions and duplications, account for a large part of the genomic diversity among individuals, and they have been implicated in many genetic diseases, including cancer in humans [8]. SVs were initially thought to be fewer in plants, but this perspective dramatically changed with the realization that almost all flowering plants are derived from multiple rounds of ancient or recent polyploidization [9]. Recently, some studies have shown that structural genome variation is closely associated with diversity in complex traits [10]. The identification of SVs has significantly evolved over the last decade from detecting large (>3 Mbp) rearrangements in metaphase spreads using light microscopy to the computational calling of SVs using genomic sequencing techniques (all ranges of SV sizes are detectable). However, it remains challenging to systematically and comprehensively analyze massive amounts of genomic data and, particularly, to detect SVs in genomes from WGS data due to computational and algorithmic limitations [11,12]. Multiple algorithms and techniques have been combined to predict and validate variants in some studies. Moreover, the repetitive content of mammalian and plant genomes makes identifying SVs challenging, especially in crops that have more repetitive sequences.

Over the last several years, heavy-ion-beam radiation has been utilized as a type of ionizing radiation, and it has been shown to induce heritable genomic deletions, duplications, insertions, translocations and SBSs in rice and *Arabidopsis* [13,14], but the effects on the genomic spectrum of soybean induced by using a heavy-ion beam have not been investigated. The soybean genome has more repetitive sequences (about 60% of the entire genome) than those of rice (about 45%) and *Arabidopsis* (about 20%), which also makes it more difficult to analyze its genomic variation. Some researchers explored and studied soybean genome variations that were induced by using a fast neutron (FN) or proton beam [15,16]. A heavy-ion beam has different physical characteristics from a proton beam or FN beam. Therefore, it is more important to clarify the mutation spectrum induced in the soybean genome using a heavy-ion beam.

Heavy-ion beams and gamma rays both ionize radiation and can release or capture electrons (called ionizations) and directly disrupt the chemical bonds of molecules when passing through matter. DNA lesions caused by ionizing radiation include nucleotide-base lesions, DNA single-strand breaks (SSBs) and DNA double-strand breaks (DSBs) [17]. As SSBs and DSBs fail to be repaired, the two mutations finally induce base-pair substitutions, INDELs and chromosome rearrangements, which are detected in later generations [18]. Heavy-ion beams have a higher rate of linear energy transfer (LET), which indicates the deposited energy of particles or photons per unit length of the target material. LET values change according to the different particles used in beams and their respective energies. Gamma rays, which comprise a type of photon, are usually considered a low-LET form of radiation. In a previous study of *Arabidopsis*, drastic and complex alterations in chromosomes were induced with a high-LET ion beam, and short INDELs appeared more easily when using a moderate-LET ion beam [19]. Similar results were also confirmed and verified in rice [20]. Copy number variations (CNVs) were detected when exposed to 4, 8, 16 and 32 Gy FN radiation; these included duplications that ranged from 499 bp to 50 Mbp in size. Hemizygous deletions in these variants ranged from 4.9 kb to 9.3 Mbp, and homozygous deletions ranged from 493 bp to 8.1 Mbp [21]. Seven mutant rice lines that were subjected to gamma rays and carbon-ion beams were compared. It was found that the number of small mutations induced by gamma-ray irradiation was higher than that induced by carbon-ion beam irradiation, and these mutations were accompanied by complex SVs [14]. These studies suggested that the structural variations induced by irradiation with different LET values were likely to be different, but attention has not been paid to the effects induced by different LETs on the phenotypic variation spectrum (in fact, in breeding, more attention is given to crop trait changes). Therefore, when analyzing a genomic spectrum, similar phenotypic compositions (representing a specific breeding outcome) in the mutation process can provide more information to help breeders select more suitable mutagens in their research.

The breeding efforts for leguminous crops are largely limited due to their narrow genetic base. The genetic variants available in the current primary soybean gene pool are poor [22]. Background mutations make it more difficult to acquire homozygotes. This lesser variation reduces the possibility of acquiring target traits. In general, depending on the type of propagule used, due consideration is paid to the appropriate strategies for mutation breeding and recovering the sought-after mutation events in homozygous states and suitable genetic backgrounds, with as few unintended mutation events as possible complicating the selection on account of linkage drag [23]. The number of mutations needed to obtain different phenotypes for breeding or theoretical research in lab, as well as the survival of mutant plants, depends on the radiation dosage used. However, variants of the same phenotype appear even if the radiation parameters, including the dosage and LET, are completely different. Because additional mutations induced by the environment are accumulated year after year, their relationship with the phenotypic composition is not obvious in later generations (after the fourth generation). All in all, during mutation breeding, any specific variation can be acquired if the size of the mutagenic population is not limited. However, the difficulty of obtaining a certain variation is different due to the variation spectra of the selected mutagens. Because a genome spectrum can provide more information that cannot be directly observed, more potential information on the phenotypic variation spectrum can be expressed by the genome spectrum in limited populations of similar phenotypic compositions. In this study, whole-genome resequencing was used to investigate soybean populations with the same phenotypic composition and different mutagenesis treatments in order to analyze the different spectra induced by the selected mutagens. The research process used in this study is displayed in Figure S1.

## 2. Results

### 2.1. Biological Effects of Carbon Ion Beam and Gamma Ray Irradiation on M_1_ Plants

**Figure 1:**
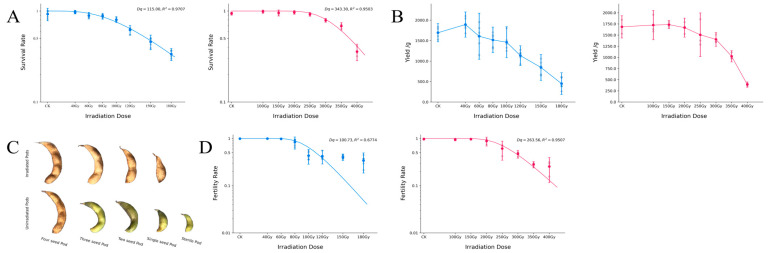
Biological effects of the carbon-ion beam and gamma-ray irradiation on M_1_ plants. The blue line represents the carbon-ion beam and the red line represents the gamma rays. (**A**) The survival rates of soybean. The continuous curve is a fitted curve (SHMT). (**B**) The yield values of soybean. (**C**) The condition of the soybean pods. (**D**) The fertility rate of soybean. The continuous curve is a fitted curve (SHMT).

The dry seeds (water content: 6.8%) were irradiated with a carbon-ion beam or gamma rays in different doses. The survival rates were investigated starting at 4 weeks after sowing (Figure 1A). In both analyses, high survival rates were maintained at low doses without significant changes, but the rates rapidly decreased at doses higher than the so-called “shoulder dose” (Dq) obtained from previous studies. The dose for 50% survival was lower when using the carbon-ion beam (142 Gy) than when using gamma-ray irradiation (376 Gy), which indicated that the negative effect of the carbon-ion beam on plant survival was greater than that of gamma rays. The RBE based on the dose for 50% survival was 2.64 between carbon-ion beam irradiation and gamma-ray irradiation. The two survival curves that were calculated for both mutagens were fitted using a single-hit multitarget (SHMT) model. The high R^2^ values of the two fitting curves indicated that the curves were well fit. The corresponding shoulder doses on each survival curve (Dq of the survival rate) were determined to be 115 Gy for carbon-ion beam irradiation and 343 Gy for gamma-ray irradiation. The RBE based on the Dq of the survival rates was 2.98 between the carbon-ion beam irradiation and gamma-ray irradiation.

The soybean yield was investigated 3 weeks after the soybeans were harvested from the field (Figure 1B). The yield of soybean after carbon-ion beam irradiation was higher than that after gamma-ray irradiation, which was mainly related to the differently planted fields. In both analyses, the highest value of the treated soybean yield did not appear in the CK group (control: nonirradiated group); rather, it appeared in the low-dose group, so this was considered a low-dose stimulation. The highest yield appeared at 40 Gy when using the carbon-ion beam, and it appeared at 100 Gy when using gamma rays. The RBE based on the highest yield was 2.5 between carbon-ion beam irradiation and gamma-ray irradiation. When the yield dropped to half of that of the control, the corresponding dose was near 120 Gy for carbon-ion beam irradiation and near 350 Gy for gamma-ray irradiation. The RBE based on the dose for the 50% yield was about 2.92 between carbon-ion beam irradiation and gamma-ray irradiation. Because of the curve’s slow descent, SHMT model fitting was not used in this analysis.

When comparing the irradiated and nonirradiated groups, the form and condition of the pods, such as the number of seeds in one soybean pod, significantly changed when the time came to harvest (Figure 1C). In the unirradiated population, most pods had three seeds; only a few pods had two or four seeds. Only a few single-seed pods were observed in some areas with weak growth, and no five-seed pods were observed during long-term planting. In the irradiated populations, sterile pods (without seeds) appeared at the end of maturity. The number of single-seed pods increased with the increase in dosage, but the total number of pods decreased. The three-seed pods almost completely disappeared in the group that was irradiated with the highest dose (Table S1). In the calculation of the fertility rate, we defined sterile pods and single-seed pods as low-fertility pods, which also indicated stunted fertility. The fertility rates of the soybeans were calculated using the ratio of low-fertility pods.

The fertility rates of the soybeans were investigated about one week before they were harvested from the field (Figure 1D). The trend of fertility was similar to that of the survival rate. The dose for a 50% fertility rate was lower when using carbon-ion beam irradiation (111 Gy) than when using gamma-ray irradiation (289 Gy). The RBE based on the dose for a 50% fertility rate was 2.60 between carbon-ion beam irradiation and gamma-ray irradiation. Compared with the survival rate, the tendency of the fertility rate was different in that the fertility rate remained much more stable at lower doses and declined faster when the dose was greater than the shoulder dose. At high doses with the carbon-ion beam, the fertility rate showed a slower downward trend, which resulted in a lower R^2^ value (0.67) for the fitted curve. However, the phenomenon of a slower downward fertility rate was weak, which led to a better R^2^ value (0.95) when using gamma rays. The fertility curves were also fitted using the SHMT model because of their similar tendencies to those of the survival rates. The Dq of the fertility rate was determined to be 101 Gy for carbon-ion beam irradiation and 263 Gy for gamma-ray irradiation. The RBE based on the Dq of the fertility rate was 2.61 between carbon-ion beam irradiation and gamma-ray irradiation.

### 2.2. Frequency and Spectrum of Phenotypic Variations in M_2_ Populations Derived from Carbon Ion Beam and Gamma Rays

**Figure 2:**
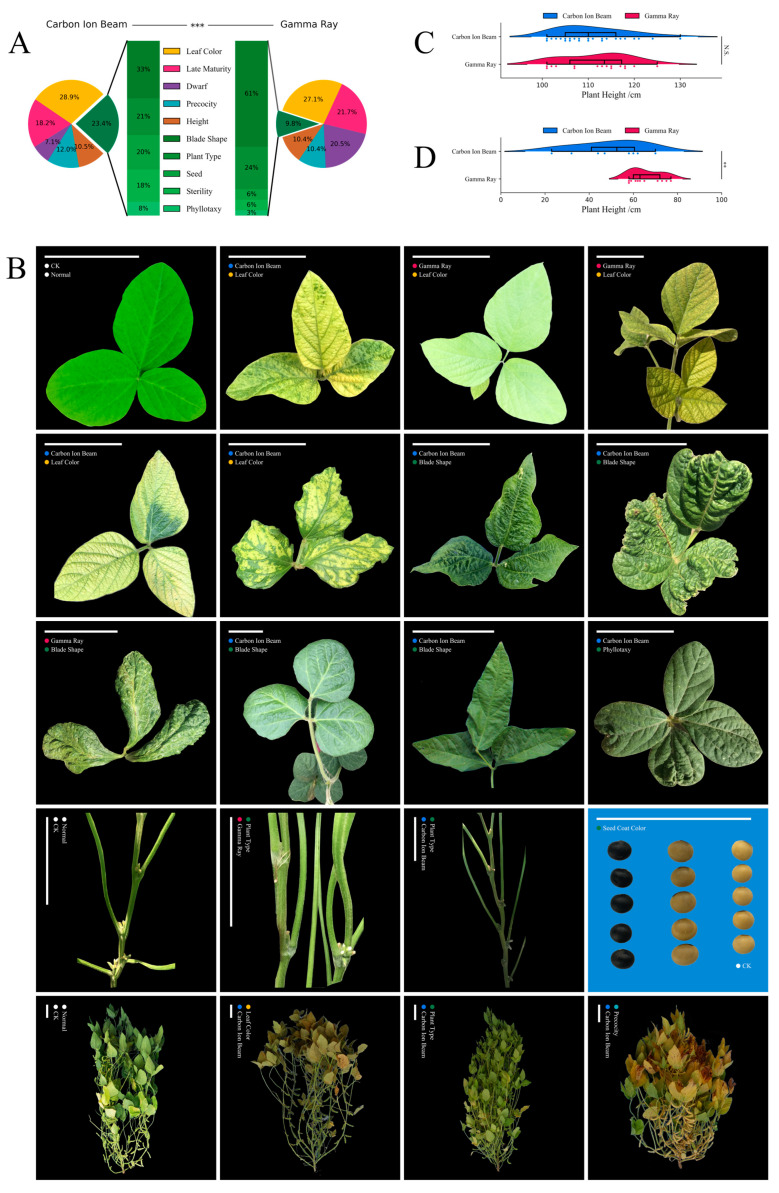
Phenotypic mutations that were identified and statistics in the M_2_ generation. (**A**) The proportion of visible mutation phenotypes in the M_2_ generation. (**B**) Phenotypes of partial mutants induced using carbon-ion-beam irradiation (blue circle) and gamma-ray irradiation (red circle); the scale bar is 10 cm. (**C**) Statistics of plant height mutation phenotypes induced using carbon-ion-beam and gamma-ray irradiation. (**D**) Statistics of the dwarf plant mutation phenotypes induced using carbon-ion-beam and gamma-ray irradiation. N.S. *p* ≥ 0.05, ** 0.001 ≤ *p* < 0.01, *** *p* < 0.001.

For each mutagen, there were about 60,000 M_2_ plants from over 2000 M_2_ families that were planted, and they were then used for mutation screening. We recorded the phenotypes and counted the number of mutation lines throughout the whole growth period. Regarding the macroscopic morphology, about 325 M_2_ families from carbon-ion-beam-irradiated populations were identified, and 336 M_2_ families from gamma-ray-irradiated populations were identified (Table 1). In the M_2_ generation, the percentage of each phenotype out of all phenotypic mutations was calculated (Figure 2A), and highly significant differences were determined using the chi-squared test. This result showed that leaf color mutations were the most frequent types in both irradiated populations (about 28.9% in the carbon-ion-beam-irradiated populations and about 27.1% in the gamma-ray-irradiated populations). Dwarf mutations occurred more frequently in the gamma-ray-irradiated populations (about 20.5% of the screened phenotypic families) than in the carbon-ion-beam-irradiated populations (about 7.1%). Infrequent mutations (colored dark green) occurred more frequently in the carbon-ion-beam-irradiated populations (about 23.4%) than in the gamma-ray-irradiated populations (about 9.8%). The infrequent mutations consisted of five subgroups, and the percentages of the five subgroups changed much more slightly with carbon-ion beam irradiation than with gamma-ray irradiation. The five subgroups with infrequent mutations were those of blade shape, plant type, seed, sterility and phyllotaxy. There were 25 blade shape mutations, 16 plant type mutations, 15 seed mutations, 14 sterility mutations and 6 phyllotaxy mutations obtained in the carbon-ion-beam-irradiated population. There were 20 blade shape mutations, 8 plant type mutations, 2 seed mutations, 2 sterility mutations and 1 phyllotaxy mutation obtained in the gamma-ray-irradiated population.

The phenotypes of the mutations were diverse, and a brief classification would omit too many details. Detailed descriptions of subphenotypic classifications and subgroups were recorded to make up for the deficiencies in the previous brief classifications (Table S2). Meanwhile, some representative phenotypic photos of the mutations were also selected to describe details that were ignored in some previous classifications (Figure 2B). Many types of leaf color mutations were detected in the M_2_ generation. Yellow leaves were the main phenotype in the leaf color mutations, and striped leaf mutations were much less common; they were only found in the carbon-ion-beam-irradiated population. The blade shape mutations also had some differences from each other, and many different styles were found in the carbon-ion-beam-irradiated population. The seed coat color was one of the seed mutation types, and a black seed coat was easier to acquire than a light-brown seed coat; the black seed coat mutations were found in both the carbon-ion-beam-irradiated population and the gamma-ray-irradiated population, but only one light-brown seed coat mutation was screened in the carbon-ion-beam-irradiated population, and it was not found when using gamma rays.

Soybean plant height is usually considered a quantitative trait; different height values can be found in one mutation line. Several of the height and dwarf mutation lines that had similar height values within the lines were selected for analysis. In the height mutation lines, no significant differences were found between the carbon-ion-beam-irradiated population and gamma-ray-irradiated population using a *t*-test (Figure 2C). However, the highest soybean mutation height value was derived from carbon-ion-beam-irradiated mutations.

In the dwarf mutation lines, a slightly significant difference was found in the carbon-ion-beam-irradiated population and gamma-ray-irradiated population using a *t*-test (Figure 2D). The height values of the dwarf mutations from the gamma-ray-irradiated population were higher than those from the carbon-ion-beam-irradiated population.

### 2.3. Overview and Classification of Sequence Variation

A total of 32 mutants with reliable phenotypes were selected for analysis and study to compare the different effects of carbon-ion beam irradiation and gamma-ray irradiation on the soybean genome. The phenotypes of the selected mutations were observed in the M_2_ generation and were further confirmed in the M_3_ generation. The whole genomes of these M_3_ plants were sequenced, of which 16 were from carbon-ion beam irradiation mutants and 16 were from gamma-ray irradiation mutants (Table S3). The coverages of all the resequenced soybean samples were more than 30×. The selected mutants had similar mutation phenotypes within their own populations, and the number of similar phenotypic mutations was the same on both sides.

**Figure 3:**
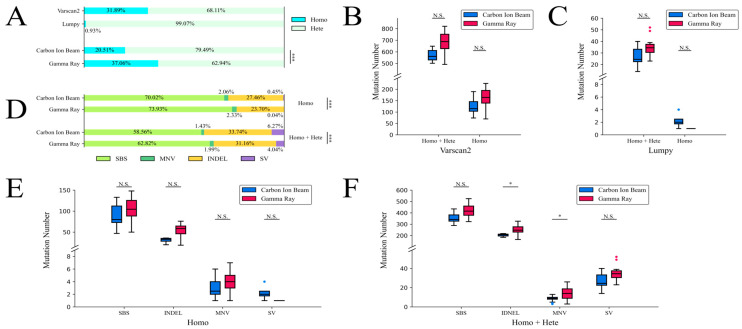
The overview and classification of the sequence variations. The homozygous and heterozygous groups are marked as Homo and Hete. (**A**) The proportion of homozygous + heterozygous sequence variants. (**B**) The number of sequence variants from Varscan2. (**C**) The number of sequence variants from Lumpy. (**D**) The proportion of sequence variants inside the variant classification. (**E**) The number of sequence variants within the variant classification of the homozygous genotype. (**F**) The number of sequence variants within the variant classification of the homozygous + heterozygous genotype. N.S. *p* ≥ 0.05, * 0.05 ≤ *p* < 0.01, *** *p* < 0.001.

After completing the necessary genome sequence alignment and basic filtering, the sequence variants were called using Varscan2 and Lumpy. The variant results of multiple samples were compared and filtered using a Python script; the details are described in the Materials and Methods section. Depending on the diploid genotype results of the detected variants, the sequence variants were divided into two groups, which were the homozygous genotype group and the heterozygous genotype group (Figure 3A). There were many fewer homozygous sequence variants (about 0.93% of the sequence variants detected using Lumpy) than heterozygous sequence variants (about 99.07% of the sequence variants detected using Lumpy) in Lumpy. The sequence variants in the same irradiated group were merged into a single file as the final variation set, and extremely significant differences were determined using a chi-squared test. In the merged group, the rates of the homozygous sequence variants (about 20.51% of the sequence variants) induced using carbon-ion-beam irradiation were lower than those (about 37.06% of the sequence variants) induced using gamma-ray irradiation. In the mutant lines, the number of detected sequence variants induced using carbon-ion-beam irradiation was lower than the number induced by gamma-ray irradiation according to the Varscan2 program, but no significant differences were determined from the *t*-test (Figure 3B). Meanwhile, the number of sequence variants detected using Lumpy also showed no significant differences according to a *t*-test. The number of sequence variants when using carbon-ion-beam irradiation was lower than that when using gamma-ray irradiation inside the homozygous + heterozygous genotype group, but the number of sequence variants when using carbon-ion-beam irradiation was greater than that when using gamma-ray irradiation inside the homozygous group (Figure 3C).

In total, 1988 and 5279 homozygous variations were induced using carbon-ion-beam irradiation and gamma-ray irradiation, respectively. For the homozygous + heterozygous genotype, 9695 and 14,243 variations were induced using carbon-ion-beam irradiation and gamma-ray irradiation, respectively. The types of sequence variations were varied. The identified sequence variations were classified into four categories, which were SBSs, INDELs, MNVs and SVs (Figure 3D). The proportions of MNVs and SVs were much smaller than those of the other categories. Highly significant differences were determined using the chi-squared test for carbon-ion-beam and gamma-ray irradiation. It could be easily seen that the SVs contributed to the main difference in the chi-squared calculation. In the mutated lines, the number of homozygous sequence variants obtained with carbon-ion-beam irradiation was lower than that obtained with gamma-ray irradiation, except for the SV type, but no significant differences were determined when using the *t*-test for each variant classification (Figure 3E). In the homozygous + heterozygous group, the number of sequence variants obtained with carbon-ion-beam irradiation was lower than that obtained with gamma-ray irradiation, but slightly significant differences were determined when using the *t*-test for the INDEL and MNV classifications (Figure 3F).

### 2.4. Characteristics of Small Sequence Variations

**Figure 4:**
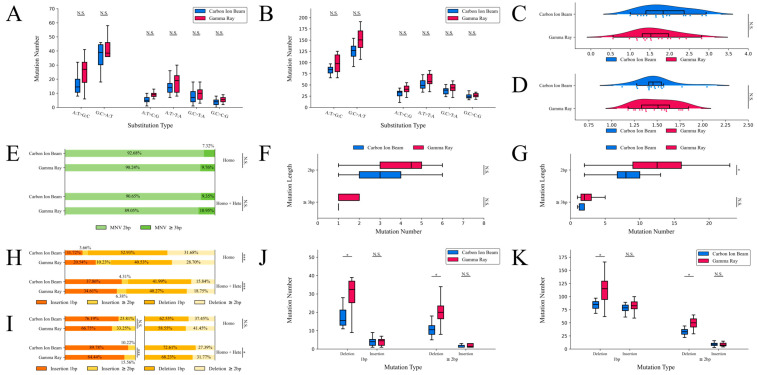
The number and characteristics of small sequence variations. The homozygous and heterozygous genotypes are marked with Homo and Hete. (**A**) The numbers of substitutions in homozygous sequence variations. (**B**) The numbers of substitutions in homozygous + heterozygous sequence variations. (**C**) The Ts/Tv ratio in the homozygous genotype. (**D**) The Ts/Tv ratio in the homozygous + heterozygous genotype. (**E**) The proportions of the MNV lengths. (**F**) The number of homozygous MNVs. (**G**) The number of homozygous + heterozygous MNVs. (**H**) The proportions of INDEL lengths. (**I**) The proportions of insertion (left) and deletion (right) lengths. (**J**) The number of INDELs in the homozygous genotype. (**K**) The number of INDELs in the homozygous + heterozygous genotype. N.S. *p* ≥ 0.05, * 0.05 ≤ *p* < 0.01, *** *p* < 0.001.

SBSs were the most abundant variants induced by both carbon-ion-beam irradiation and gamma-ray irradiation (Figure 3D). Two classifications—transition mutations and transversion mutations—were identified for both mutagens. In most of the mutant lines, more transitions than transversions were induced by both mutagens. A total of 1392 SBSs for carbon-ion-beam irradiation and 3903 SBSs for gamma-ray irradiation were identified in the homozygous genotype, and the numbers of SBSs in the mutant lines were identified and classified (Figure 4A). A total of 5677 SBSs for carbon-ion-beam irradiation and 8947 SBSs for gamma-ray irradiation were identified in the homozygous + heterozygous genotype, and the numbers of SBSs in the mutant lines were identified and classified (Figure 4B). No significant differences in the different types of substitutions were determined in the homozygous or homozygous + heterozygous genotype. The transition-to-transversion (Ts/Tv) ratios of the mutant lines were recorded; they were 1.88 for carbon-ion-beam irradiation and 1.63 for gamma-ray irradiation in the homozygous genotype (Figure 4C). In the homozygous + heterozygous genotype, the ratios were 1.48 for carbon-ion-beam irradiation and 1.50 for gamma-ray irradiation, and no significant differences were determined in the homozygous or homozygous + heterozygous genotype (Figure 4D).

MNVs are clustered mutations. MNVs have received increasing attention in recent sequence variation analyses. The total number of MNVs was divided into two classifications according to their length. In both the homozygous + heterozygous and homozygous genotypes, a higher proportion of MNVs greater than 3 bp was detected in the gamma-ray-irradiated group than in the carbon-ion-beam-irradiated group, but no significant differences were observed between the carbon-ion-beam irradiation and gamma-ray irradiation groups (Figure 4E). In all, 41 MNVs in the carbon-ion-beam-irradiated group and 123 MNVs in the gamma-ray-irradiated group were identified in the homozygous genotype, and the numbers of MNVs in the mutant lines were identified and classified (Figure 4F). In total, 139 additional MNVs from the carbon-ion-beam irradiation group and 283 additional MNVs from the gamma-ray irradiation group were identified in the homozygous + heterozygous genotype, and the numbers of MNVs in the mutant lines were identified and classified (Figure 4G). A significant difference in 2 bp MNVs was observed between the carbon-ion-beam irradiation and gamma-ray irradiation group regarding the homozygous + heterozygous genotype.

The total number of INDELs was divided into two categories—insertion mutations and deletion mutations—and each of these two categories was then divided into two classifications according to their length. Extremely significant proportional differences were determined using the chi-squared test for the four classifications inside the homozygous and homozygous + heterozygous genotypes (Figure 4H). The proportions of insertions and deletions are displayed separately (Figure 4I). The result of the chi-squared test for the homozygous + heterozygous group showed that an extremely significant difference was determined in the insertion category between the two mutagens, and a slightly significant difference was determined in the deletion category between the two mutagens. In total, 462 deletions and 84 insertions after carbon-ion-beam irradiation and 866 deletions and 385 insertions after gamma-ray irradiation were identified in the homozygous genotype, and the number of INDELs in the mutant lines was identified and classified for each classification (Figure 4J). A further 1892 deletions and 1379 insertions for carbon-ion-beam irradiation and 2619 deletions and 1819 insertions for gamma-ray irradiation were identified in the homozygous + heterozygous genotype, and the number of INDELs in the mutant lines was identified and classified for each classification (Figure 4K). Significant differences in deletions between the carbon-ion beam and gamma-ray irradiation were determined using a *t*-test for the homozygous and homozygous + heterozygous genotypes.

### 2.5. Characteristics of Structural Variations

**Figure 5:**
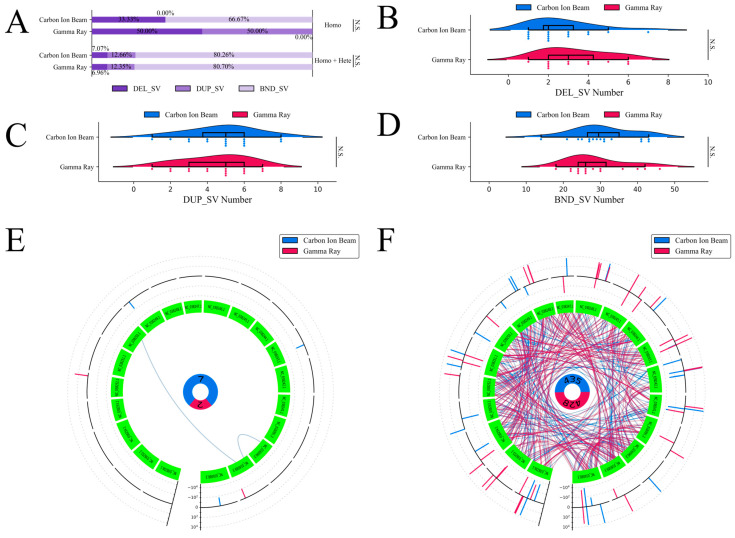
The number and characteristics of structural variations. Deletions over 50 bp, duplications over 50 bp and breakends are marked with DEL_SV, DUP_SV and BND_SV. The homozygous and heterozygous genotypes are marked with Homo and Hete. (**A**) The proportions of different SV types. (**B**) The number of DEL_SVs in the homozygous + heterozygous genotype. (**C**) The number of DUP_SVs in the homozygous + heterozygous genotype. (**D**) The number of BND_SVs in the homozygous + heterozygous genotype. (**E**) The distribution of SVs on the chromosomes in the homozygous genotype. (**F**) The distribution of SVs on each chromosome in the homozygous + heterozygous genotype. N.S. *p* ≥ 0.05.

Unlike small sequence variations, the detection of structural variations is considered unreliable. However, these results may, to some extent, provide us with some information on the differences between the carbon-ion beam and gamma-ray irradiation. Three classifications—marked with DEL_SV, DUP_SV and BND_SV—were identified for both mutagens (Figure 5A). DEL_SV and DUP_SV refer to deletions and duplications whose length was over 50 bp. No significant differences in the proportions of SVs were observed between the two physical mutagens for either the homozygous or homozygous + heterozygous genotype. For the homozygous genotype, three DEL_SVs, no DUP_SVs and six BND_SVs were identified after carbon-ion-beam irradiation, and one DEL_SV, one DUP_SV and no BND_SVs were identified after gamma-ray irradiation. In total, 43 DEL_SVs were identified after carbon-ion-beam irradiation, and 40 DEL_SVs were identified after gamma-ray irradiation in the homozygous + heterozygous genotype; the number of DEL_SVs in the mutant lines was identified and classified (Figure 5B). A further 77 DUP_SVs were identified from carbon-ion-beam irradiation, and 71 DUP_SVs were identified from gamma-ray irradiation in the homozygous + heterozygous genotype; the number of DUP_SVs in the mutant lines was identified and classified (Figure 5C). A total of 488 BND_SVs were identified from carbon-ion-beam irradiation, and 464 BND_SVs were identified from gamma-ray irradiation in the homozygous + heterozygous genotype; the number of BND_SVs in the mutant lines was identified and classified (Figure 5D).

There were 1171 scaffolds in the soybean reference genome that failed to accurately assemble the 20 chromosomes. Some SVs, especially BND_SVs, had a relationship with the unassembled scaffolds, and these SVs were filtered. For the homozygous + heterozygous genotype, seven filtered SVs were identified from carbon-ion-beam irradiation, and two filtered SVs were identified from gamma-ray irradiation. The positions of the filtered SVs on the chromosome were identified and recorded (Figure 5E). For the homozygous + heterozygous genotype, a further 438 filtered SVs were identified from carbon-ion-beam irradiation, and 428 filtered SVs were identified from gamma-ray irradiation; the positions of the filtered SVs on the chromosome were identified and recorded (Figure 5F).

### 2.6. Impacts of Mutations on Genes

**Figure 6:**
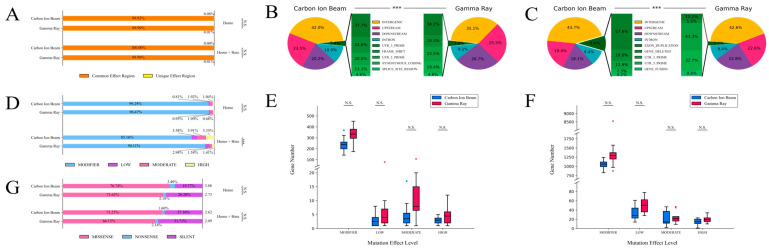
The proportions and characteristics of gene effects. The homozygous and heterozygous genotypes are marked with Homo and Hete. (**A**) The proportions of selected genes in commonly affected regions. (**B**) The proportions of genes in commonly affected regions due to homozygous sequence variations. (**C**) The proportions of genes in commonly affected regions due to homozygous + heterozygous sequence variations. (**D**) The proportions of gene effect levels. (**E**) The numbers of gene effects inside each effect level in the homozygous genotype. (**F**) The numbers of gene effects inside each effect level in the homozygous + heterozygous genotype. (**G**) The proportions of functional classes for changed codons. N.S. *p* ≥ 0.05, *** *p* < 0.001.

All of the detected mutations were analyzed via SnpEff, which involved the use of a self-built database (based on GCA_000004515.4) to predict the possible effects of the mutations on the genes. The distribution of the regions of the affected genes was not exactly the same in both mutagens. The number of genes in commonly affected regions was selected to compare the two mutagens, and the proportion of the selected gene effects was over 99% (Figure 6A). No significant differences were observed in either the homozygous or homozygous + heterozygous genotype. The genes in the commonly affected regions that were affected by mutations in the homozygous genotype were selected to calculate the percentages of the types of effects of both mutagens (Figure 6B). The results showed that the intergenic region was the most frequent region for both types of irradiation, and the percentage of this affected region (about 42.0%) was relatively higher with carbon-ion-beam irradiation than with gamma-ray irradiation (about 35.1%). The number of gene effects for the downstream region was greater with gamma-ray irradiation (about 26.7%) than with carbon-ion-beam irradiation (about 20.2%). Extremely significant differences were determined using the chi-squared test between the two mutagens for the homozygous genotype. The genes in the commonly affected regions that were affected by mutations in the homozygous + heterozygous genotype were selected to calculate the percentages for both mutagens (Figure 6C). The results showed that the intergenic region was also the most frequent region, but the percentages were close in both mutagens. The gene proportion of effects for the downstream region was greater with gamma-ray irradiation (about 22.8%) than with carbon-ion-beam irradiation (about 18.1%). In particular, the proportion of gene effects for the exon duplication region was far greater with carbon-ion-beam irradiation (about 5.41%) than with gamma-ray irradiation (about 0.27%). When using the chi-squared test, a highly significant difference was also determined between the two mutagens in the homozygous + heterozygous genotype.

The sequence variations were divided into four classifications according to the genes’ effect levels (Figure 6D). Although this classification was not strictly related to phenotypes, it could provide some clues as to the sequence variations. Four classifications—modifier, low, moderate and high—were identified for both mutagens. It was easily found that the proportions of these four classifications had differences between the two mutagens for the homozygous + heterozygous genotype, and an extremely significant difference was determined using the chi-squared test. In total, 3810 modifier variations, whereby 32 variations were classified as low, 76 were classified as moderate and 41 were classified as high were identified for the carbon-ion-beam irradiation, and 12,144 modifier variations, whereby 119 variations were classified as low, 239 were classified as moderate and 86 were classified as high were identified for the gamma-ray irradiation for the homozygous genotype; the numbers of these four effect levels in the mutant lines were recorded (Figure 6E). However, no significant differences were determined in each category of the genes’ effect levels when using a *t*-test between the two mutagens. A total of 16,814 modifier variations, whereby 707 variations were classified as low, 1166 were classified as moderate and 1057 were classified as high were identified for the carbon-ion-beam irradiation, and 27,400 modifier variations, whereby 857 variations were classified as low, 448 were classified as moderate and 410 were classified as high were identified for the gamma-ray irradiation for the homozygous + heterozygous genotype; the numbers of these four effect levels in the mutant lines were recorded (Figure 6F). No significant differences were determined between the two mutagens in each category of the genes’ effect levels using a *t*-test.

Three types of partial SBSs were recognized based on their effects on the genome: missense SBSs, nonsense SBSs and silent SBSs (Figure 6G). For the homozygous genotype, 66 missense SBSs, 3 nonsense SBSs and 17 silent SBSs were identified for carbon-ion-beam irradiation, and 223 missense SBSs, 5 nonsense SBSs and 85 silent SBSs were identified for gamma-ray irradiation in the homozygous genotype. The ratio of missense SBSs to silent SBSs was 3.88 when using carbon-ion beam irradiation, and it was 2.73 when using gamma-ray irradiation. For the homozygous + heterozygous genotype, 164 missense SBSs, 5 nonsense SBSs and 60 silent SBSs were identified for carbon-ion beam irradiation, and 340 missense SBSs, 11 nonsense SBSs and 163 silent SBSs were identified for gamma-ray irradiation in the homozygous genotype. The ratio of missense SBSs to silent SBSs was 2.62 when using carbon-ion beam irradiation, and it was 2.09 when using gamma-ray irradiation. For the homozygous and homozygous + heterozygous genotypes, the ratios between the missense SBSs and silent SBSs were all smaller with gamma-ray irradiation than with carbon-ion beam irradiation, but no significant differences were determined between the two mutagens using the chi-squared test.

## 3. Discussion

With the development of mutagenesis technology, the methods of physical mutagenesis have gradually developed, which implies that it is necessary to select more appropriate physical mutagenesis methods in the process of mutation breeding to cope with different research and application needs. It is of great significance to compare and analyze the similarities and differences between physical mutagenesis methods in breeding processes.

### 3.1. Biological Effects of Carbon Ion Beam Irradiation and Gamma Ray Irradiation on Soybean

In long-term breeding research and practice, determining the optimal mutagenesis dose for plants or crops according to the biological effects on the M_1_ generation is considered effective. In recent years, increasing attention has been paid to finding the optimal dose of mutagens in breeding using different biological indicators. The optimal dose of mutagenesis in plant material is affected by the source of the mutagenesis and the type of plant or crop material [20,24]. The survival rate is the most widely used biological effect indicator in breeding. It is well known that mutation screening is a labor-intensive task since it requires a large population of M_2_ plants (the number of individuals needed for mutation screening); if the mutations occur at a low frequency, a huge population size may be required. The size of the mutation screening population is influenced by various factors, including, but not limited to, survival rate, fertility rate, mutation frequency and characteristics of the plant’s genome. In recent years, the fertility rate and plant height have been used as biological effect indicators in plants to determine the optimal dose of mutagenesis [25]. In addition, increasing attention has been paid to finding the optimal dose of mutagens in breeding by combining or using different biological indicators [24,26].

In this study, the biological effects of two mutagens on the survival, fertility and yield of soybean were measured. In previous studies, the fertility and yield of the M_1_ generation were given little attention. The SHMT model was used to analyze the survival and fertility rates in the M_1_ generation in order to obtain the corresponding shoulder doses for each indicator. These doses are considered very important points as they enable more effective mutations in the genome with a relatively weak impact on propagation [27,28]. More materials with variable phenotypes are needed for breeding and genetic research. The abundance of mutant resources in mutagenesis is directly determined by the number of M_1_ plant seeds. There is no doubt that the fertility and yield of the M_1_ generation can provide information on the population size of the M_2_ generation (the screened generation). The shoulder dose for fertility was lower than that for the survival rate for both mutagens, indicating that fertility was a more sensitive indicator than survival and that irradiated plant seeds began to decline at a very rapid rate, which resulted in the loss of many seeds that contained mutations, which could also be confirmed in the results for the yield. Therefore, a dose ranging from the Dq of fertility to the Dq of the survival rate might be the best dose for mutation breeding.

Different mutagens produce the same biological effects with different doses, and such differences were described using the RBE [29]. At present, no research has described statistics or analyses of the RBE of soybean fertility in the M_1_ generation. It was observed that the number of seeds in soybean pods significantly changed as the radiation dose increased, as previously described. However, it is important to display the RBEs of different biological indicators as they might still depend on other biological indicators. In this study, the RBEs in several selected effect points were calculated, with values ranging from 2.5 to 3.0. In particular, the RBEs of fertility based on the selected biological effect points were very close, which potentially indicated similar trends in the fertility rate between the two mutagens. The close trends in the fertility rate for the two mutagens meant that the fertility was likely comparable to that in theoretical studies.

In summary, when considering the optimal dose, we should consider not only the survival of the plant but also the ability of the plant to produce seeds. In our research, we suggested that the optimal dose was from the Dq of fertility to the Dq of survival. Thus, the optimal dose of the carbon-ion beam was 101 Gy–115 Gy, and the optimal dose of the gamma rays was 263 Gy–343 Gy.

### 3.2. Phenotypic Spectra of Carbon-Ion-Beam Irradiation and Gamma-Ray Irradiation in Soybean

The relationship between the irradiation dose and frequency of phenotypic mutations in the M_2_ generation was analyzed in rice and *Arabidopsis thaliana* [30,31]. Recently, phenotypic diversity has become increasingly important in breeding, and a wider mutation spectrum (more types of mutation) has been found to have great potential to facilitate successful breeding. The most prevalent mutation type induced was that of dwarf variants in *Capsicum annuum*, which was induced using gamma-ray irradiation [32]. Several mutants displayed no pigments on their leaves and stems or trichomes on their leaves; these were isolated from *Arabidopsis thaliana*, which was irradiated using a carbon-ion beam [33]. Only ten salt-tolerant maize lines derived from gamma-ray irradiation were isolated from over 2000 plants [34]. Although the criteria for screening phenotypes varied slightly among different studies, these results still prove that different mutation phenotypes occur with different frequencies. Although the frequencies of phenotypic mutations may indicate differences in the complete mutant spectra obtained with different mutagens, it is almost impossible to obtain a complete mutation spectrum for a mutagen.

In this study, the phenotypic mutation profile, which included type and frequency, was investigated in the M_2_ generation. The total number of phenotypic mutant families in the carbon-ion-beam-irradiated population was very close to the number in the gamma-ray-irradiated population. The phenotype of the largest percentage was also the same for both mutagens, and the proportions of low-frequency phenotypic mutations were different in the populations subjected to the different mutagens. These results indicate that the highest-frequency phenotype was easily obtained using either the carbon-ion beam or gamma rays, but the low-frequency phenotypes were not the same in the two mutagens.

There were more dwarf mutant lines obtained with gamma-ray irradiation than with carbon-ion beam irradiation, but the mutant lines with the highest and lowest plant heights were found to come from carbon-ion-beam-induced mutations. Our results also showed that the mutation ability of carbon-ion beam irradiation was much stronger than that of gamma-ray irradiation. Unlike the appearance of phenotypic variations, plant height was characterized by continuous values. Mutations with different plant heights could be screened in either the carbon-ion-beam-irradiated population or gamma-ray-irradiated population. Although dwarf mutant lines could be more easily obtained (in a higher proportion) using gamma rays, the extreme plant height variations all occurred in the carbon-ion-beam-irradiated population. These extreme plant height variations could also be considered low-frequency variations.

In summary, the results for phenotypic variations may imply that the variations in the spectra of mutagens were mainly manifested in their abilities to acquire low-frequency variations. Unfortunately, we cannot indefinitely plant mutagenic seeds to obtain a “complete” variation spectrum, so it is difficult to compare the frequency of low-frequency variations in a population of a limited size.

### 3.3. Genomic Mutations Induced Using Carbon-Ion-Beam Irradiation and Gamma-Ray Irradiation

The LET is one of the major physical parameters used to distinguish between different types of ionizing radiation, and it was reported to be closely related to DNA damage repair and genomic variation [13,35]. Genomic variation is the source of phenotypic variation in mutagenesis breeding. Proton beam irradiation always induces a higher number of clustered DNA lesions than gamma-ray irradiation does, regardless of the proton energy [36]. More deletions (≥2 bp) and SVs were discovered when using carbon-ion beam irradiation [37]. Argon-ion-beam irradiation with a high LET value induced a greater number of rearrangements and large deletions (≥100 bp) than carbon-ion beam irradiation did [19]. Gamma-ray irradiation tended to induce larger numbers of small mutations than carbon-ion beam irradiation did; SVs were considered the specific characteristics of the mutations induced using carbon-ion beam irradiation [14]. The frequency of MNVs induced using gamma-ray irradiation was approximately 2.35 times the frequency of those induced using carbon-ion beam irradiation without any SVs for the two mutagens [38]. These results suggest that the proportions of types of genomic variation may be responsible for the LET value.

In this study, the identified sequence variations were classified into four categories, namely, SBSs, INDELs, MNVs and SVs. In particular, the length of the INDELs was not more than 50 bp, and the SVs containing deletions with a length of over 50 bp were named DEL_SV. SBSs were the most abundant type of mutation induced by both mutagens, though gamma-ray irradiation induced more SBSs than carbon-ion beam irradiation, which was also observed in a study of mutational effects on *Arabidopsis* seedlings [39]. However, SBSs were detected relatively more frequently with low-LET radiation than with high-LET radiation; this finding is similar to the conclusions drawn by a study on *Arabidopsis* seedlings [37]. The average Ts/Tv ratios with carbon-ion beam irradiation and gamma-ray irradiation in the homozygous genotype were 1.88 and 1.63, respectively. A similar result for the Ts/Tv ratio with gamma-ray irradiation, which was lower than that with carbon-ion beam irradiation, was reported in rice [38]. However, it was the opposite for the homozygous + heterozygous genotype. In comparison, this conclusion was probably inaccurate because the Ts/Tv values in every single sample were recorded, and no significant differences were found between the two mutagens. MNVs are substitution mutations that occur on successive bases, and these MNVs can be produced in tandem within several helical coils of DNA using ionizing radiation [40,41]. In this research, MNVs were detected after both carbon-ion beam irradiation and gamma-ray irradiation. Gamma-ray irradiation induced more MNVs than carbon-ion beam irradiation did, and the same result was found in rice [38]. The increased number of MNVs was attributed to the greater indirect effects of gamma-ray irradiation than carbon-ion beam irradiation.

In most cases, INDELs contained insertions and deletions of any length, but in this article, in order to distinguish insertions and deletions of large fragments in SVs, the INDELs were referred to as insertions and deletions of no more than 50 bp. INDELs have always been a highly important form of sequence variation in genome variation, and more deletions are thought to be a characteristic of mutations in a genome obtained using a high LET [28,42]. In this study, a similar result was found in the homozygous genotype, where the carbon-ion beam could induce a higher percentage of deletions than gamma-ray irradiation could. However, in terms of the homozygous + heterozygous genotype, the percentage of deletions obtained with carbon-ion beam irradiation was not greater than that obtained with gamma-ray irradiation, which might have been due to the greater number of DNA repairs because direct damage was unlikely to produce insertions, and DNA repair can produce insertions. The proportion of deletions and insertions of no less than 2 bp obtained with carbon-ion beam irradiation was lower than that obtained with gamma-ray irradiation, but the difference was determined only in the homozygous + heterozygous genotype, which possibly means that the ability to produce ≥2 bp deletions was not enhanced compared with gamma-ray irradiation. However, the higher percentage of deletions obtained with carbon-ion beam irradiation resulted in an increase in the number of ≥2 bp deletions, which was also reported in other research on gamma-ray irradiation [37].

With the development of sequencing technology and sequence analysis technology, sequence variations in large fragments of a genome have attracted more and more attention. Some studies have shown that SVs have a great effect on phenotypes. SVs can be frequently induced by carbon-ion beam irradiation in chrysanthemum [35] and carnation [43]. In this study, the SVs detected in the homozygous + heterozygous genotype were used to perform a comparative analysis as the number of homozygous SVs was too small. The SVs were divided into three types, namely, DEL_SVs, DUP_SVs and BND_SVs. There were no significant differences in the SV ratios between the two mutagens. The numbers of DEL_SVs, DUP_SVs and BND_SVs in each mutation line were also similar in both mutagens. In particular, between carbon-ion beam and gamma-ray irradiation, Lumpy detected slightly more SVs, while Varscan2 detected the opposite number of SBSs, INDELs and MNVs. In general, the numbers and proportions of the three types of SVs were similar in both mutagens, but the proportion of SV with respect to the total sequence variations detected when using carbon-ion beam irradiation was higher than when using gamma-ray irradiation.

### 3.4. Mutation Effects Induced Using Carbon-Ion-Beam Irradiation and Gamma-Ray Irradiation

The SVs made up the main difference in the sequence variations induced by the two mutagens; SVs not only directly affect the copy number or structure of genes, but they may also change the genomic landscape in relation to the transcription patterns of genes in specific genomic areas [44]. In research on biological theory, greater sequence variation in the target phenotype makes it more difficult to identify target genes. SVs were found to change important traits in tomatoes by modifying gene expression levels and dosage [45]. In this study, the gene effects of all genomic variations that included SVs were analyzed. Compared with gamma-ray irradiation, a higher proportion of homozygous variations in the intergenic regions was retained when using carbon-ion beam irradiation, though this was not seen in the homozygous + heterozygous genotype. This result may imply that the genomic variations induced by carbon-ion beam irradiation were more likely to form homozygotes. In the homozygous + heterozygous genotype group, many more variations in the exon duplication regions were retained when using carbon-ion beam irradiation. For all kinds of genomic variations in the homozygous + heterozygous genotype group, the proportion or number of SVs was the main difference between the carbon-ion beam and gamma rays, so the SVs might have had a close relationship with the effects on the exon duplication region. Direct damage by physical mutagens does not generate insertions or duplications, which shows that carbon-ion beam irradiation might induce a different DNA repair pathway, which might lead to more variations in the exon region. The percentage of high effects obtained with carbon-ion beam irradiation was higher than that obtained with gamma-ray irradiation, which might imply that the mutations induced by the carbon-ion beam had a stronger effect on the genes. This, in turn, might mean that carbon-ion beams can affect genes more effectively. The higher proportion of nonsense SBSs obtained with gamma-ray irradiation means there were more target mutations and unexpressed proteins, while the higher proportion of missense SBSs obtained with carbon-ion beam irradiation showed that it could effectively change the amino acid sequences and thus increase the possibility of protein improvement. This might have been due to the molecular principle that more low-frequency phenotypic variations were obtained in the carbon-ion-beam-irradiated population than in the gamma-ray-irradiated population.

## 4. Materials and Methods

### 4.1. Plant Materials and Irradiation Conditions

The dried seeds of Williams 82 (*Glycine max* (L.) Merr.) were exposed to carbon-ion beams or gamma rays. The seeds were placed inside a 35 mm culture dish, with the soybean seeds’ umbilici pointing upward. A carbon-ion beam (energy: 80 MeV/nucleon; average LET inside seeds: 30 keV/µm) was generated at the Heavy Ion Research Facility in Lanzhou (HIRFL), and gamma rays (source: ^137^Cs; average LET inside seeds: 0.2 keV/µm) were generated at the Hefei Institutes of Physical Science (HFIPS). The doses set for the carbon-ion beam were 40, 60, 80, 100, 120, 150 and 180 Gy, and the doses set for the gamma ray was 100, 150, 200, 250, 300, 350 and 400 Gy. For each dose, about 20–23 seeds were used for irradiation. The irradiated soybean seeds were sown in a field located in Baiyin (Gansu, China, 103.73° E, 36.03° N).

### 4.2. Analysis of the Effects of Irradiation on the M_1_ Generation

The ratio of biological effects was used to fit the curves (usually used for survival curves), which were drawn based on the single-hit multitarget theory (SHMT) using the following equation, as previously described:

$$Fitted\;Rate=1-{\left(1-e^{-D/D_{0}}\right)}^{m}$$

where *e* is the natural constant, *D* is the dose, *D*_0_ is the dose conferring a 37% biological effect and *m* is an extrapolated number for the parameters. The data were fitted with the least squares method using Python scripts. The shoulder dose (*Dq*) of the fitted curves was calculated with the following equation:

$$Dq=D_{0}\times \ln m$$

where ln (mathematical operation symbol) is the natural logarithm, *D*_0_ is the dose conferring a 37% biological effect and *m* is an extrapolated number for the parameters.

The survival rate was determined 5 weeks after the seeds were sown in a field. Five replications of more than 20 seeds were used for each dose of the carbon-ion beam, and three replications of more than 20 seeds were used for each dose of the gamma rays. To calculate the survival rate, repeated average data were used for the final results, and then curve fitting was performed. The LD50 dose (where 50% of the plants died) and the shoulder dose (*Dq* of the survival rate) were obtained from the curve of the survival rate and irradiation dose.

The fertility rate was determined before the plants were harvested from the field. Five replications of three plants’ data were collected for each dose of the carbon-ion beam, and three replications of three plants’ data were collected for each dose of gamma rays. To calculate the fertility rate, we used the following equation:

$$Fertility\;Rate=\frac{total\;pods-low\;fertility\;pods}{total\;pods}\times 100\%$$

Low-fertility pods included sterile pods and single-seed pods. To calculate the fertility rate, repeated average data were used for the final results, and then curve fitting was performed. The dose for a 50% fertility rate and the shoulder dose (*Dq* of the fertility rate) were obtained from the curve of the fertility rate and irradiation dose.

The yield was determined 3 weeks after the plants were harvested from the field when the plants were sufficiently dry. Five replications of all plants in similar areas were collected for each dose of the carbon-ion beam, and three replications of all plants were collected for each dose of the gamma rays. To facilitate the calculation of the yield, all plant materials were planted on land with similar areas. The ratio of the yield of all soybeans in a single area to that of the control group was used as the final yield result.

### 4.3. Mutant Screening Using Phenotypes in the M_2_ Generation and Genetic Stability Confirmation in the M_3_ Generation

All of the soybean seeds were planted in the breeding field. M_2_ seeds, which were obtained via the individual harvest method in the M_1_ generation, were collected and sowed using the plant-to-row method. Rows that had candidate mutations (plants with visible phenotypic changes throughout the whole growth period when compared to the wild type) were recorded. M_3_ seeds, which were also obtained via the individual harvest method in the M_2_ generation, were collected and then sown using the plant-to-row method. The phenotypes of each row were recorded, compared and matched with the M_2_ generation. Finally, the most reliable soybean mutations were determined.

### 4.4. Whole Genome Sequencing and Analysis of DNA Mutations

Thirty-four individuals with different mutant phenotypes were selected from the M_3_ lines. Sixteen of them had been subjected to gamma-ray irradiation, and another sixteen had been subjected to carbon-ion beam irradiation, in addition to another two that came from the original seeds that were subjected to a similar method, including the same sowing, harvest and environmental conditions, but they were not irradiated. Genomic DNA was extracted from the dried leaves of each individual plant using a cetyltrimethylammonium bromide (CTAB)-based method [46]. Genomic DNA sequencing was performed to obtain paired-end reads (150 bp) by using the Illumina Novaseq 6000 platform at Novogene Technologies Company (Shanghai, China). For sequence preprocessing, low-quality reads with an adaptor sequence were removed from the raw sequences. The cleaned reads were mapped to the soybean assembly as the reference genome (RefSeq Assembly Accession: GCF_000004515.5) using the BWA (0.7.17) [47] to generate files in the BAM format. Qualimap2 (0.7.17) [48] was used to evaluate the alignment data. SBSs and small INDELs between the reference genome and the mapped sequences from each sample were called from the BAM-format files using samtools (4.1.7) [49], and then the sequence mutations were filtered using Varscan2 (4.23) [50] on a pipeline. To detect the SVs, duplicate sequences were removed from the BAM-format files using Picard MarkDuplicates [51]. Then, Lumpy (1.0.1) [52] was used to detect SVs with the default settings. The genotyping results of each sample were combined using svtools (0.4.0) [53].

The SBSs, INDELs and SVs that were common between the CK individuals but polymorphic between the wild-type individuals and a single M_3_ line were selected and used for the analysis. All of the mutations with low-quality values were filtered, and the mutations that were commonly detected in two or more M_3_ lines were excluded. Varscan2 provided allele frequency (AF) values for the SBSs and INDELs detected in each sample. For the SBSs and INDELs, the variants of AF ranging from 25% to 75% were called heterozygous mutations, and those with an AF ≥ 75% were regarded as homozygous mutations. For the SVs, the homozygous or heterozygous SV mutations were determined by genotype (GT), as reported by the Lumpy algorithms. The variants of the GT that had only one zero were called heterozygous mutations, and those with a GT that had no zeroes were regarded as homozygous mutations.

In particular, if some of the detected SBSs had contiguous locations on the reference genome, these SBSs were incorporated into MNVs using Python scripts. SBSs and MNVs were analyzed as two independent variant types. The SBSs that made up the MNVs were not involved in the calculation of the Ts/Tv ratios. This was the same strategy that SnpEff used to calculate the Ts/Tv ratios. However, the software specifications of SnpEff (4.3t) [54] do not emphasize this strategy, which was discovered in its open-source code.

After the above steps, some of the output mutations were verified using IGV (2.8.13) [55]. All the mutations were annotated using SnpEff.

### 4.5. Statistical Analysis

At least three replicates were used for each treatment, and the data are presented as mean ± 1.97 ×standard deviation (mean ± 1.97 × SD). The number and proportion of each category of mutations induced by gamma rays were compared with those induced by the carbon-ion beam using Student’s *t*-test and a chi-squared analysis, and the significance level was 0.05. The third-party drawing library Matplotlib [56] was used to draw all of the statistical charts.

## 5. Conclusions

In breeding processes in previous studies, a dose that was slightly lower than the Dq of the survival rate was considered the best dose for breeding, and the Dq of the fertility rate was also slightly lower than the Dq of the survival rate in this article. The range of RBEs that included fertility between the two mutagens was about 2.5–3.0 in the M_1_ generation. We suggest that the optimal dose of carbon-ion beams for soybean mutation breeding is 101 Gy–115 Gy, and the optimal dose of gamma rays is 263 Gy–343 Gy. In the progeny of both mutagens, slightly fewer phenotypic mutations were obtained using carbon-ion beam irradiation, and these were accompanied by more low-frequency phenotypic mutations; this result might have been due to the higher proportion of missense gene effects or the higher proportion of SVs induced by the carbon-ion beam. The total number of genomic variations induced by the carbon-ion beam was lower than those induced by gamma rays; this has the potential to alleviate problems caused by linkage drag in soybean mutation breeding. Meanwhile, many more SVs were detected when using carbon-ion-beam irradiation, which could lead to more exon duplications. There were more missense gene effects after carbon-ion-beam irradiation, and there were more nonsense gene effects after gamma-ray irradiation, which meant that the changes in amino acid sequences were different between the carbon-ion beam and gamma rays. Based on these results, mutagenic breeding with carbon-ion beams might be able to meet the new demands in breeding and theoretical research. For industrial usage in mutation breeding, it is better to use carbon-ion beams to obtain low-frequency phenotypes, fewer background genomic mutations and mutations with higher proportions of SVs. Due to the high cost of carbon-ion beams, other mutagens should be chosen to realize other breeding goals in mutation breeding.

## Figures and Tables

**Table 1:** The number of screened phenotypic mutations in M_2_ generation.

Phenotype	Carbon Ion Beam	Gamma Ray
Leaf Color	94	91
Late Maturity	59	73
Dwarf	23	69
Precocity	39	35
Height	34	35
Other (infrequent)	76	33

## Data Availability

All the NGS data files are available in the CNCB (publicly accessible at https://ngdc.cncb.ac.cn/gsa (accessed on 11 May 2023)), and the Bio-Project accession number is PRJCA016265. Other datasets used and/or analyzed during the current study are available from the corresponding author on reasonable request.

## Supplementary Materials

The following supporting information can be downloaded at https://www.mdpi.com/article/10.3390/ijms24108825/s1.
