# Supplementary figures and images for: Comparison and Characterization of Phenotypic and Genomic Mutations Induced by a Carbon-Ion Beam and Gamma-ray Irradiation in Soybean (Glycine max (L.) Merr.)
Source: Int J Mol Sci. 2023 May 16;24(10):8825. doi: 10.3390/ijms24108825 (PMC10218375; doi:10.3390/ijms24108825)

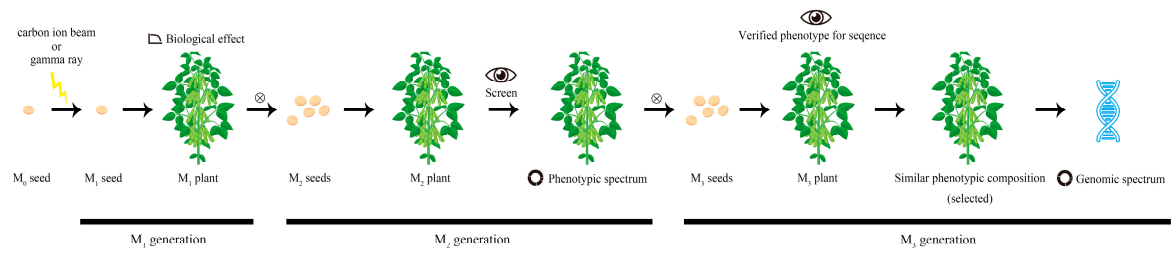

**Figure S1.** The research process of this article.

Supplement: Supplementary file 1 [file ijms-24-08825-s001.zip › Figure S1.pdf]

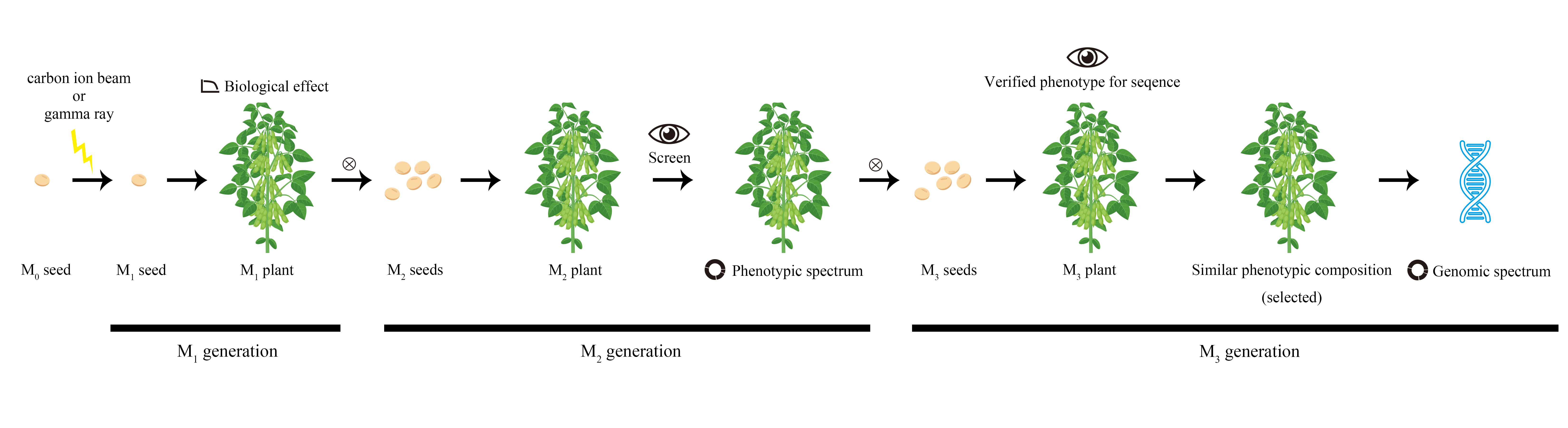

Supplement: Supplementary file 1 [file ijms-24-08825-s001.zip › Figure S1.png]
